# Eyedrop Administration of DPP-4 Inhibitors: A New Strategy for Treating Early Stages of Diabetic Retinal Disease

**DOI:** 10.3390/ijms27104361

**Published:** 2026-05-14

**Authors:** Hugo Ramos, Olga Simó-Servat, Cristina Hernández, Rafael Simó

**Affiliations:** 1 Diabetes and Metabolism Research Unit, Vall d’Hebron Research Institute (VHIR), 08035 Barcelona, Spain; olga.simo@vallhebron.cat (O.S.-S.); cristina.hernandez@vhir.org (C.H.); 2 Center for Networked Biomedical Research of Diabetes and Associated Metabolic Diseases (CIBERDEM), Carlos III Health Institute (ICSIII), 28029 Madrid, Spain; 3 Department of Medicine, Autonomous University of Barcelona, 08193 Barcelona, Spain

**Keywords:** diabetes, diabetic retinopathy, diabetic retinal disease, neurodegeneration, neuroprotection, eyedrops, topical administration, DPP-4 inhibitors, sitagliptin, GLP-1

## Abstract

This review is intended to highlight the need for non-invasive and earlier therapies for diabetic retinal disease (DRD), one of the most common complications of diabetes, with a high and increasing socioeconomic burden. Due to the growing evidence regarding the key role of neurodegeneration in the earliest stages of the disease and the underlying pathophysiological mechanisms, the relevance of evaluating the potential efficacy of neuroprotective therapies is emphasized. More specifically, the review addresses the current state of a promising neuroprotective approach based on the inhibition of the enzyme dipeptidyl peptidase-4 (DPP-4) using specific inhibitors administered via eyedrops, which allow direct retinal action on the neurovascular unit. The review discusses the main preclinical findings of a therapeutic strategy based on one DPP-4 inhibitor, sitagliptin, against early DRD in different experimental animal models and in vitro studies. In summary, sitagliptin eyedrops exhibit neuroprotective, anti-inflammatory, and antioxidant properties while reducing glial activation, hyperpermeability of the blood–retinal barrier, and the formation of acellular capillaries, leading to a functional improvement of the diabetic retina. However, as sitagliptin efficacy has only been evaluated at the preclinical level, clinical studies are needed to validate the translational applicability and long-term efficacy of topical administration not only of sitagliptin but also of other DPP-4 inhibitors for treating retinal diseases in which neurodegeneration plays a pathogenic role.

## 1. Introduction

In recent years, research in the field of diabetic retinal disease (DRD), one of the most prevalent complications of diabetes, has focused on the identification of effective, non-invasive therapies with minimal adverse effects that can target the earliest stages of the disease [1]. These early stages constitute a significant unmet medical need, as currently available treatments, including intravitreal injections of corticosteroids or vascular endothelial growth factor (VEGF) inhibitors, as well as laser photocoagulation, are primarily directed at advanced stages, when visual impairment is already present [2,3]. Given that approximately one-third of individuals with diabetes will develop DRD and that the global incidence of diabetes is ceaselessly increasing, the disease represents a substantial and growing socioeconomic burden for healthcare systems worldwide [4].

DRD has traditionally been defined as a microvascular complication; nonetheless, growing evidence indicates that neurodegeneration plays a pivotal role during the earliest stages of the disease, even prior to the appearance of detectable signs. In accordance with this paradigm shift, the American Diabetes Association redefined DRD as a highly specific neurovascular complication [5]. These early alterations are characterized by dysfunction of the retinal neurovascular unit (NVU), a highly integrated and specialized functional coupling composed of retinal neurons, glial cells (including Müller cells, astrocytes, and microglia), and vascular components, such as endothelial cells and pericytes, which together maintain retinal homeostasis and vascular integrity [6,7,8].

The increasing understanding of early NVU impairment and the role of neurodegeneration at this stage has opened new avenues for the development of therapeutic strategies based on neuroprotection to address the unmet medical need that early DRD represents [9,10,11,12]. This review aims to briefly describe the early neurodegenerative processes that take place in the retina, together with some of the current experimental neuroprotective therapies, with a special focus on dipeptidyl peptidase-4 inhibitors (DPP-4i) eyedrops, a promising approach that has been extensively investigated by our group at the preclinical level [13,14,15,16,17,18].

## 2. The Key Role of Neurodegeneration in Early Diabetic Retinopathy

Retinal neurodegeneration is increasingly recognized as a fundamental and early component of DRD [8,10]. Growing evidence indicates that functional and structural alterations in retinal neurons occur at very early stages of the disease, even preceding clinically detectable microvascular abnormalities [8,10,19]. Multiple neuronal populations, including retinal ganglion cells, amacrine cells, bipolar cells, and photoreceptors, as well as Müller glial cells, are affected during this early phase [20,21,22].

Chronic hyperglycemia represents the main initiating event in early neuronal impairment. The excess of intracellular glucose enhances glycolytic flux and activates four major metabolic pathways: protein kinase C (PKC), hexosamine, polyol, and advanced glycation end-product (AGE) pathways [23]. The activation of these mechanisms disrupts cellular homeostasis through oxidative stress, mitochondrial dysfunction, osmotic imbalance, abnormal protein modifications, and pro-inflammatory signaling [23,24]. Collectively, these alterations compromise synaptic integrity and neuronal survival.

Oxidative stress is a pivotal downstream mechanism [23,24,25]. Hyperglycemia increases mitochondrial superoxide production through the overactivation of the electron transport chain and the consequent electron leakage [23,24,26], which results in the production of cytosolic reactive oxygen species (ROS) via nicotinamide adenine dinucleotide phosphate (NADPH) oxidases (NOX), particularly NOX2 [25,27]. Concurrently, antioxidant defenses are impaired, partly due to dysregulation of transcriptional responses mediated by nuclear factor erythroid 2-related factor 2 (NRF2) [28,29]. The resulting redox imbalance leads to mitochondrial damage, accumulation of dysfunctional organelles, and further amplification of oxidative injury [24,28].

Glutamate excitotoxicity constitutes another key contributor to neurodegeneration [30,31]. Impaired glutamate uptake by Müller cells, associated with reduced expression of glutamate transporters, leads to extracellular glutamate accumulation and overstimulation of ionotropic receptors, particularly in retinal ganglion cells [30,31,32]. Additionally, diabetes disrupts presynaptic protein expression and axonal transport, further impairing synaptic transmission [16,33].

Inflammatory processes are tightly interconnected with metabolic and oxidative stress [34]. Hyperglycemia-induced activation of microglia and reactive gliosis of Müller cells and astrocytes promote the release of pro-inflammatory cytokines, including tumor necrosis factor alpha (TNF-α) and interleukin-1 beta (IL-1β), as well as additional ROS [34,35,36]. This pro-inflammatory environment exacerbates neuronal injury and contributes to the early neurodegenerative changes [34].

Diabetes has also been associated with reduced retinal availability of several neurotrophic and neuroprotective factors, including pigment epithelium-derived factor (PEDF), somatostatin (SST), interstitial retinol-binding protein (IRBP), and glucagon-like peptide-1 (GLP-1) [37,38,39,40]. Although certain factors such as vascular endothelial growth factor (VEGF) and erythropoietin (EPO) are upregulated [41,42], the overall predominance of the downregulation of the neurotrophic support promotes progressive neuronal degeneration.

Finally, dysfunction of the NVU involves both neuronal and vascular impairments [43,44]. Endothelial damage and pericyte loss disrupt blood–retinal barrier (BRB) integrity, leading to vascular leakage, impaired autoregulation, and hypoxia [43,44,45]. In turn, neuronal stress promotes the release of vasoactive and pro-apoptotic mediators that further damage vascular components, establishing a self-perpetuating cycle of neurovascular deterioration [46].

In summary, chronic hyperglycemia initiates a complex network of metabolic, oxidative, inflammatory, excitotoxic, and neurovascular alterations that lead to early retinal neurodegeneration. Neuronal and vascular dysfunctions amplify each other, which justifies the new consideration of DRD as a progressive neurovascular disease rather than an exclusively microvascular complication of diabetes.

## 3. Neuroprotective Therapeutic Strategies Against Early Diabetic Retinopathy

The recognition of neurodegeneration as an early event in the pathophysiology of DRD has led to the development of novel experimental neuroprotective therapies [12]. The different strategies proposed and evaluated target the multiple mechanisms underlying retinal neurodegeneration, including oxidative stress, excitotoxicity, synaptic damage, inflammation, glial activation, neurovascular interplay, and the reduction in endogenous neuroprotective factors [11,12,47].

### 3.1. Antioxidants and Mitochondrial Protectors

Oxidative stress and mitochondrial dysfunction play central roles in retinal neuronal injury [25,26,48]. Classical antioxidants such as α-lipoic acid improve mitochondrial function and reduce oxidative damage [49,50], while mitochondria-targeted compounds like SkQ1 prevent ganglion cell loss [51]. Activation of antioxidant pathways dependent on nuclear factor erythroid 2-related factor 2 (NRF2) (e.g., sulforaphane) restores redox balance and reduces apoptosis [52]. All these strategies aim not only to scavenge ROS but also to reestablish mitochondrial homeostasis.

### 3.2. Reduction in Excitotoxicity and Synaptic Preservation

Glutamate-mediated excitotoxicity is a major driver of neuronal loss in diabetes [53,54]. Brimonidine reduces glutamate release and activates pro-survival pathways (PI3K/Akt, ERK), demonstrating functional preservation in early DRD in the EUROCONDOR trial [55,56]. Citicoline stabilizes neuronal membranes and enhances bioenergetics, improving retinal electrophysiological responses [57,58]. NMDA receptor antagonists such as memantine attenuate calcium influx and excitotoxic damage, although systemic adverse effects have limited clinical translation [59]. Collectively, these therapies aim to maintain synaptic integrity and prevent early neuronal dysfunction.

### 3.3. Anti-Inflammatory and Immunomodulatory Agents

Inflammation amplifies neurodegenerative cascades in DRD [34]. SOCS1-derived peptides inhibit JAK/STAT signaling and reduce glial activation and vascular leakage [60]. Minocycline modulates microglial activation and decreases pro-inflammatory cytokines [61], while palmitoylethanolamide suppresses glial reactivity via PPAR-α activation. Inhibition of P2X7 receptors further reduces inflammasome activation and neuronal apoptosis [62]. These findings highlight inflammation control as a relevant neuroprotective strategy.

### 3.4. Neurotrophic and Growth Factor-Based Therapies

Loss of neurotrophic support is a hallmark of DRD and contributes substantially to retinal neuronal vulnerability and apoptosis. Treatments aimed at restoring the levels/functionality of neurotrophic factors that are reduced in the diabetic retina, such as somatostatin (SST), ciliary neurotrophic factor (CNTF), brain-derived neurotrophic factor (BDNF), pigment epithelium-derived factor (PEDF), nerve growth factor (NGF), and erythropoietin (EPO), have all demonstrated the ability to promote retinal ganglion cell survival and reduce apoptosis at the preclinical level [63,64,65,66]. In addition, glucagon-like peptide-1 (GLP-1), an incretin hormone known for its neuroprotective effects in the central nervous system (CNS), and dipeptidyl peptidase-4 (DPP-4) are expressed in the retina and participate in retinal GLP-1/GLP-1 receptor (GLP-1R) signaling axis [14,16,17,37,67,68]. Accumulating evidence provides the rationale for a more detailed discussion of GLP-1 signaling and DPP-4 inhibition as therapeutic neuroprotective strategies in DRD.

## 4. DPP-4 Inhibition via Eyedrops: An Emerging Neuroprotective Approach in Early Diabetic Retinopathy

### 4.1. GLP-1 Peptide: An Incretin with Neuroprotective Effects in the Central Nervous System

The GLP-1 peptide is a hormone that plays an important role in the regulation of glucose homeostasis. It is mainly produced by intestinal enteroendocrine L-cells in response to food intake and contributes to the lowering of postprandial glucose levels. GLP-1 functions as an incretin, meaning it enhances the release of insulin in response to glucose. It also inhibits the absorption of glucose after food intake by delaying gastric emptying and reducing intestinal motility. Furthermore, GLP-1 suppresses glucagon release and decreases hepatic glucose production [69]. Through its receptors (GLP-1R) in the brainstem and hypothalamus, GLP-1 also promotes satiety, leading to a reduction in food and water intake [70]. These glucoregulatory properties have positioned GLP-1-based therapies (liraglutide, semaglutide, dulaglutide, etc.) as a highly effective in type 2 diabetes. In addition, the major additional effect of these therapies on body weight reduction has placed them at the forefront of anti-obesity pharmacotherapy [71].

The GLP-1 peptide is derived from the pre-proglucagon gene and is enzymatically cleaved and modified to generate the biologically active forms of GLP-1, namely, GLP-1 (7–36) amide and GLP-1 (7–37). These forms of GLP-1 are the primary circulating bioactive molecules in humans [72]. GLP-1 mediates its effects by binding to GLP-1R, inducing a conformational change that triggers receptor activation. Briefly, this activation promotes the dissociation of an inhibitory G-protein and the association of a stimulatory G-protein, which in turn activate adenylyl cyclase at the plasma membrane. As a result, ATP is converted into cyclic adenosine monophosphate (cAMP), which serves as a second messenger, triggering the activation of protein kinase A and the consequent intracellular signaling events [73].

To a lesser extent, GLP-1 is also synthesized from pre-proglucagon by neurons located in the solitary tract nucleus of the brainstem, giving rise to two distinct sources of GLP-1 in the body [74]. Some evidence suggests that GLP-1 released by the gut and GLP-1 produced within the brain constitute separate systems with distinct functions [75,76]. While gut-derived GLP-1 contributes more to the functions previously mentioned, brain-derived GLP-1 acts more as a neurotransmitter or a neuromodulator. Nonetheless, the specific interplay between these two systems remains uncertain because the ability of GLP-1 to cross the blood–brain barrier (BBB) makes it difficult to distinguish which functions are attributable to gut or brain-derived GLP-1 [75,76]. However, the local production of GLP-1 in the brain emphasizes its relevance in the CNS, where its functions are mainly neuroprotective in nature, as it promotes neuronal differentiation and survival [77], regulation of synaptic plasticity, and neurogenesis [78,79].

Deficiency in GLP-1 or a malfunction of this signaling pathway has been associated with multiple CNS neurodegenerative diseases, such as Parkinson’s disease (PD) or Alzheimer’s disease (AD) [80]. In these neurodegenerative disorders, administration of GLP-1R agonists has been shown to preserve neuronal survival and function in experimental models. Significant improvements have been reported in relevant pathological features, including motor deficits, striatal dopaminergic dysfunction, and neuronal loss in PD, as well as glutamatergic neuronal loss and cognitive decline in AD. Beneficial effects have also been observed not only in experimental models but also in early-phase clinical trials involving patients with AD and PD [81]. Collectively, these findings underscore the substantial therapeutic potential of GLP-1-based therapies as pharmacological strategies for the treatment of neurodegenerative diseases.

### 4.2. The Beneficial Effects of GLP-1 in the Diabetic Retina

Given that the retina is ontogenetically derived from the brain [82], our group hypothesized that GLP-1 could also be useful in preventing diabetes-induced retinal neurodegeneration [37]. In fact, we and others have identified the presence of GLP-1 and GLP-1R in the human retina [37,83]. Additionally, some studies have revealed reduced levels of GLP-1 and GLP-1R in the retina of diabetic patients [37,84]. These findings suggest that GLP-1/GLP-1R exerts a physiological neuroprotective role in healthy retina, but its diabetes-induced downregulation could be involved in the development or neurodegeneration that occurs in the early stages of DRD.

GLP-1 and GLP-1R agonists administered through intravitreal injections or topical application have displayed neuroprotective properties in various experimental studies focused on DRD [37,67,68,85]. They have exhibited positive effects on reactive gliosis, apoptosis, inflammation, and functional abnormalities in the retina. Mechanistically, GLP-1 receptor activation inhibits the accumulation of glutamate, impedes the upregulation of proapoptotic markers, reduces the levels of ROS and pro-inflammatory cytokines, and promotes the activation of survival pathways [37,67,68,86,87]. The vasculotropic effects of GLP-1R activation encompass the protection of the BRB against diabetes-induced vascular leakage by maintaining the expression of tight junction proteins and downregulating factors such as VEGF and IL-1β [37,67]. Importantly, when GLP-1 or GLP-1RAs are topically or intravitreally administered, this is due to direct action within the retina, independent of any improvement in blood glucose levels. By contrast, when GLP-1RAs are systemically administered, it becomes challenging to differentiate whether the observed effects are attributable to blood glucose control or a direct action within the eye [85,88,89,90].

### 4.3. DPP-4 Inhibitors: An Alternative Therapeutic Approach Based on GLP-1R Activation

The plasma half-life of the active forms of GLP-1 is relatively short, lasting approximately 1–2 min, due to their high susceptibility to the catalytic activity of the DPP-4 enzyme. This enzyme cleaves off the first two N-terminal amino acids of the peptide, resulting in the formation of the GLP-1 metabolite 9–36 amide. However, this metabolite is unable to activate the GLP-1R receptor. Additional cleavages by neutral endopeptidase 24.1 give rise to smaller and also inactive metabolites [91]. These observations led to the hypothesis that DPP-4 enzyme inhibition, by means of increasing the half-life of GLP-1, could be a therapeutic alternative to improve glucose metabolism-related diseases. At present, there are five different DPP4 inhibitors (DPP-4i) on the market that have been approved by the Food and Drug Administration (FDA) or the European Medicines Agency (EMA) for oral administration for the treatment of type 2 diabetes mellitus in adults [92]. The first DPP-4i, sitagliptin, was approved by the FDA in 2006, and the second, saxagliptin, was approved three years later. Later, linagliptin and alogliptin were approved. Vildagliptin was approved in 2007 by the EMA, but not the FDA [92]. There exist some differences among them related to both molecular structure and mechanism of action. On the one hand, alogliptin, linagliptin, and sitagliptin belong to the xanthine class, forming noncovalent bonds with DPP-4. On the other hand, saxagliptin and vildagliptin are cyanopyrrolidines that establish a covalent bond with the active site serine. However, despite these differences, all share the common ability to decrease the degradation of GLP-1 [93].

The well-established neuroprotective effects of GLP-1 in CNS diseases, together with the demonstrated safety of DPP-4i, have led to the emergence of DPP-4i as a promising new approach in preclinical studies for the treatment of CNS diseases. In experimental AD, they have shown promising results by reducing amyloid beta accumulation, neuronal cell apoptosis, tau hyperphosphorylation, mitochondrial dysfunction, oxidative stress, and neuroinflammation, leading to a cognitive improvement [94,95]. Additionally, in experimental PD, DPP-4i abrogated oxidative stress and the pro-inflammatory and proapoptotic environment of the substantia nigra, increased striatal dopamine levels, and reduced nigral neuronal loss [96,97]. Furthermore, in a model of premature aging, this group of drugs has exhibited the ability to prevent hippocampal neuronal cell death and mitigate impaired cognitive function [98].

### 4.4. The Beneficial Effects of Topical Administration of DPP-4 Inhibitors in the Diabetic Retina

It should be emphasized that most DPP-4i, including sitagliptin, are unable to cross the BRB [99,100], and for this reason, most of the systematic reviews and meta-analyses on the systemic (oral) administration of DPP-4i have reported a neutral effect on DRD [101,102,103].

Given the significant role of intraretinal downregulation of GLP-1 in the pathophysiology of DRD and the encouraging outcomes of experimental treatments using topical ocular administration of GLP-1 and GLP-1R agonists in diabetic retinas, it seems reasonable to consider DPP-4i a therapeutic approach for DRD based on GLP-1 enhancement. However, the presence of DPP-4 has been reported in the human retina, and it is particularly abundant in retinal pigment epithelium (RPE) [13]. In addition, a higher content of DPP-4 was found in the RPE of diabetic donors in comparison with non-diabetic donors [13]. High levels of DPP-4 in the RPE could reduce the effectiveness of topical GLP-1 administrations, as all drugs that reach the retina via the transscleral route are first challenged by the choroid and the RPE [104]. Therefore, preservation of endogenous retinal GLP-1 through topical DPP-4i, such as sitagliptin, which reaches the retina at a pharmacological concentration by the transscleral route in approximately 15 min after topical administration [105], has been proposed as a rational therapeutic strategy for early DRD [13,14,16,17,18]. This is important information for understanding the rationale for using topical (i.e., eyedrops) administration of DPP-4i. However, it is worth mentioning that both the route of absorption and pharmacokinetic data need further validation, as only one preclinical study using just sitagliptin has been performed until now [105].

It is worth mentioning that DPP-4i has other effects unrelated to the enhancement of GLP-1. In this regard, in the absence of GLP-1, sitagliptin and linagliptin protected against TNF-α-mediated inflammation in cultures of retinal bovine endothelial cells and human endothelial cells, respectively [106,107]. In addition, linagliptin displayed neuroprotective effects in a model of hyperglycemia-induced neurodegeneration in *Caenorhabditis elegans* in which GLP-1R is not produced [108]. Furthermore, DPP-4i also inhibits the degradation of other peptides, including neuropeptide Y (NPY), peptide YY (PYY), gastric inhibitory polypeptide (GIP), chemokine (C-C motif) ligand 5 (CCL5), and SDF-1 [109]. This is important because, for instance, NPY is downregulated in DRD [110], and the rescue of the NPY levels by DPP-4i could exhibit potential neuroprotective effects, such as the attenuation of NMDA-induced apoptosis in retinal neurons and preservation of the integrity of inner retinal vasculature [111]. Therefore, it remains challenging to distinguish which effects of DPP-4 inhibition are mediated by GLP-1R-dependent mechanisms and which occur independently of GLP-1 signaling. Differentiating the actions mediated exclusively by GLP-1 from those driven by other DPP-4 substrates, as well as their potential synergistic interactions, requires further investigation. In this context, in vitro studies in RPE and retinal endothelial cells show that sitagliptin reduces hyperpermeability under diabetic-like conditions in the absence of GLP-1 in the culture medium and without endogenous production, supporting GLP-1-independent effects on the BRB [112].

Another benefit of using DPP-4i is its superior stability and higher cost-effectiveness compared to GLP-1R agonists. Although the actual cost may differ based on variables such as insurance coverage and geographical location, DPP-4i is frequently accessible in generic versions, rendering it more economically accessible when contrasted with branded GLP-1R agonists [113].

In summary, the lower cost, greater stability, and GLP-1R-independent mechanisms of DPP-4i strengthened the hypothesis that topical administration of DDP-4i provides additional benefits beyond those of GLP-1R agonists [113], thereby increasing the scientific interest in its experimental investigation and clinical translation.

#### 4.4.1. Preservation of Retinal Neurotransmission: A Primary Mechanism of Neuroprotection of Sitagliptin Eyedrops

A transcriptomic profiling performed on diabetic (db/db) mice treated topically with sitagliptin revealed that the major effect of DPP-4 inhibition in the diabetic retina is the preservation of key molecular programs that modulate synaptic integrity and neurotransmission [17]. Using a combined approach that included microarray analysis, gene set enrichment analysis (GSEA), and subsequent validation by RT-qPCR and protein assessments, sitagliptin was shown to inhibit the downregulation at the mRNA and protein levels of critical proteins involved in synapse formation, axonal transport, vesicle trafficking, and neurotransmitter release [16,17]. Rather than acting on isolated targets, sitagliptin appeared to stabilize an integrated synaptic network at both structural and functional levels.

Among the most significantly differentiated genes were *C1ql1*, which encodes a secreted protein implicated in synaptogenesis and synaptic maintenance [114], and *Kif1b* and *Kif1bp*, encoding components of the KIF1B–KBP complex. This complex is essential for axonal elongation and for the transport of synaptic vesicle precursors and other cargoes along microtubules [115]. Given that kinesin family proteins are crucial for maintaining axonal integrity and that their dysfunction has been linked to axonal degeneration, the preservation of *Kif1b*/*Kif1bp* expression strongly suggests protection of neuronal connectivity. In parallel, the maintenance of *C1ql1* expression may contribute to sustained synaptic stabilization and prevention of synapse loss, a hallmark of early diabetic neurodegeneration [17].

GSEA further revealed significant positive enrichment of multiple biological pathways related to synaptic transmission, including transmission across chemical synapses, neurotransmitter transport, vesicle-mediated transport in synapse, regulation of postsynaptic membrane potential, and axon guidance [17]. These findings indicate that sitagliptin preserves coordinated biological processes related to neuronal communication rather than exerting a limited effect restricted to a single specific pathway.

At the presynaptic level, sitagliptin maintained the expression of core elements of the soluble NSF attachment protein receptor (SNARE) machinery responsible for vesicle docking and fusion, including SNAP-25, syntaxin-1 (*Stx1a*), and synaptobrevin/VAMP2. It also preserved regulatory proteins, such as Munc-13 (*Unc13a*) and Munc-18 isoforms (*Stxbp2*, *Stxbp4*, *Stxbp6*), which orchestrate SNARE complex assembly and vesicle priming. In addition, vesicle-associated proteins involved in stabilization, mobilization, and calcium-dependent exocytosis, such as synaptophysin, synapsin I, synaptotagmin 1, and SV2B release [116,117,118,119,120,121,122], were significantly preserved [16,17]. Sitagliptin also maintained expression of VGLUT1 (*Slc17a7*), a key transporter responsible for glutamate loading into synaptic vesicle DRD [123]. Considering that diabetes is associated with reduced levels of several of these proteins, leading to impaired vesicle release and electrophysiological dysfunction [124,125], their preservation likely contributes to improvements in retinal functional responses, which will be discussed in greater detail later.

Importantly, sitagliptin also exerted marked effects at the postsynaptic compartment [17]. Treatment prevented the downregulation of NMDA receptor subunits (*Grin1*, *Grin2b*, *Grin2d*) and the AMPA receptor subunit Gria1, both of which are central regulators of excitatory synaptic transmission and plasticity [126,127]. In addition, scaffolding proteins of the postsynaptic density, including PSD-95 (*Dlg4*), PSD-93 (*Dlg2*), and CASK [128,129], were preserved [17]. These proteins regulate receptor clustering, synaptic architecture, and downstream signaling integration [128,129]. Given that dysregulation of glutamatergic signaling and postsynaptic destabilization contribute to excitotoxic stress and neurodegenerative progression in diabetic retinas, maintenance of these elements suggests that sitagliptin supports both structural integrity and functional responsiveness of postsynaptic neurons.

Nagamatsu et al. already reported that oral sitagliptin was able to increase syntaxin 1 clusters in the plasma membranes of pancreatic beta cells from db/db mice, promoting vesicle docking and insulin exocytosis [130], while Kutsyr et al. showed the capacity of oral sitagliptin to preserve presynaptic and postsynaptic elements in experimental retinitis pigmentosa [131].

Immunofluorescence studies also demonstrated that the protective effects of sitagliptin extend across both the inner and outer plexiform layers, indicating widespread preservation of retinal synapses [16,17]. This wide distribution suggests that the drug preserves synaptic connectivity in multiple neuronal populations, including bipolar, amacrine, and ganglion cells, rather than acting only on a specific synaptic subtype.

A substantial proportion of these neuroprotective effects may be mediated by activation of the retinal GLP-1/GLP-1R axis secondary to DPP-4 inhibition and increased endogenous GLP-1 availability. In fact, GLP-1R activation has been shown to modulate both GABAergic and glutamatergic transmission and to attenuate glutamate-induced excitotoxicity [132,133]. However, GLP-1-independent mechanisms cannot be excluded. DPP-4 is expressed in neuronal compartments and has been detected in association with presynaptic proteins, suggesting a potential direct role in synaptic physiology [134]. Moreover, additional DPP-4 substrates involved in synaptic modulation may contribute to the overall neuroprotective profile observed [135,136,137].

Collectively, these findings indicate that sitagliptin eyedrops preserve the molecular architecture and the functionality of both pre- and postsynaptic compartments in experimental DRD. Additionally, sitagliptin stabilizes synaptic protein networks, maintains axonal transport systems, and supports receptor organization, leading to the inhibition of early synaptic dysfunction and neuronal impairment, thus targeting a central component of diabetic retinal neurodegeneration.

#### 4.4.2. The Antioxidant Properties of Sitagliptin Eyedrops

Beyond the main neuroprotective effect of sitagliptin eyedrops, studies in 12-week-old db/db mice revealed the high antioxidant capacity of the compound [14]. This diabetic model is characterized by an enhanced ROS generation, marked by an increase in superoxide (O_2_•^−^) production and a significant oxidative damage to DNA/RNA and proteins, together with a compromised antioxidant defense system, where the NRF2 pathway and several major antioxidant enzymes, including GPX, GR, CuZnSOD, MnSOD, and CAT, are downregulated [14,138,139,140,141]. Topical sitagliptin effectively counteracted this redox imbalance. The treatment reduced superoxide levels and significantly attenuated oxidative damage to nucleic acids and proteins. At the molecular level, sitagliptin preserved both mRNA and protein expression of key antioxidant components, including NRF2 and its downstream enzymatic defenses (GPX, GR, CuZnSOD, and MnSOD) [14]. In addition, sitagliptin reduced the number of TXNIP-positive cells [14]. Given that TXNIP inhibits thioredoxin and promotes oxidative stress and apoptosis [142,143], its downregulation represents an additional mechanism by which sitagliptin may restore retinal redox homeostasis.

These findings are consistent with previous reports demonstrating activation of the NRF2/ARE pathway by systemic administration of sitagliptin. For instance, in β-amyloid-induced AD models, sitagliptin enhanced NRF2 signaling and improved antioxidant defenses [144]. Similarly, increased activity of antioxidant enzymes, such as SOD, has been described independently of glucose lowering [145]. However, most prior studies relied on systemic administration, making it difficult to distinguish between direct antioxidant effects and indirect improvements secondary to better glycemic control. In contrast, the topical administration approach supports local retinal action, providing stronger evidence for a direct antioxidative effect within the diabetic retina.

Diabetes induces the upregulation of PKC-δ and PKC-β isoforms, which are implicated in ROS amplification, NF-κB activation, and vascular dysfunction [48]. This upregulation was prevented by topical sitagliptin [14]. These findings are aligned with reports showing that other DPP-4i, such as saxagliptin, reduce PKC hyperactivation in non-retinal tissues, suggesting that modulation of PKC signaling may be a broader class effect [146]. It remains to be clarified whether PKC normalization is secondary to ROS reduction or whether it is directly influenced by DPP-4 inhibition, but the simultaneous improvement in both pathways indicates a general stabilization of redox signaling.

Notably, in the TRPV2^+/−^ rat, a non-diabetic model of retinal neurovascular unit dysfunction characterized by progressive neurodegenerative and vascular alterations closely similar to those observed in diabetes, eyedrops of sitagliptin also exerted antioxidant effects [18]. Specifically, a reduction in retinal levels of 8-hydroxyguanosine, indicating decreased oxidative DNA/RNA damage, and partial prevention of gene downregulation of antioxidant enzymes, such as SOD1 and SOD2, were observed [18]. These results support the ability of topical sitagliptin to modulate retinal oxidative stress independently of the diabetic milieu.

These antioxidant effects could be explained by the increased levels of GLP-1, which may contribute to NRF2 activation; however, the antioxidant profile of sitagliptin does not appear to be identical to the antioxidant properties of GLP-1 eyedrops. Despite topical GLP-1 reducing oxidative damage in the same experimental model, sitagliptin demonstrated additional effects, such as the restoration of the protein levels of GPX, GR, and CuZnSOD [14,68]. Moreover, antioxidant effects of DPP-4i have been reported in GLP-1-free in vitro systems, further supporting the existence of GLP-1-independent mechanisms.

Altogether, topical sitagliptin restores the balance between prooxidant and antioxidant mediators in early diabetic retina through preservation of the NRF2 axis, maintenance of enzymatic defenses, reduction in TXNIP expression, and normalization of PKC signaling, reducing DNA/RNA and protein damage.

#### 4.4.3. The Anti-Inflammatory Effects of Sitagliptin Eyedrops

Topical administration of sitagliptin has been shown to exert a clear anti-inflammatory effect at the retinal level. Treatment with sitagliptin eyedrops reduced IκBα phosphorylation and the subsequent translocation of NF-κB to the nucleus [14,147]. As a consequence, a significant decrease in the pro-inflammatory cytokines IL-1β and TNF-α was observed [14]. These findings indicate that sitagliptin is able to modulate the IκBα/NF-κB signaling pathway locally in the retina.

In the same line, previous in vitro studies have reported that sitagliptin reduces NF-κB activation in stimulated endothelial cells in the absence of GLP-1 in the cell medium [148]. Additionally, other DPP-4i, such as linagliptin, have been shown to decrease TNF-α accumulation and NF-κB activation in cultures of retinal endothelial cells [106,107]. Altogether, these data support that the anti-inflammatory effects of DPP-4 inhibition may occur independently of GLP-1 enhancement.

Regarding the potential mechanisms involved, a microarray analysis identified *Commd8* as one of the most differentially expressed genes after sitagliptin treatment [147]. Since this gene is related to IκBα turnover, it could be hypothesized that its modulation contributes to the reduction in NF-κB activation. Recent evidence suggests that COMMD8 plays a role in immune cell migration and humoral responses [149]. Therefore, its downregulation might have anti-inflammatory implications beyond the NF-κB pathway.

Additionally, sitagliptin treatment significantly reduced VCAM-1 expression at both mRNA and protein levels. VCAM-1 is an important adhesion molecule involved in leukocyte recruitment and vascular inflammation [150]. Interestingly, this reduction further supports the anti-inflammatory properties of sitagliptin.

Furthermore, similar anti-inflammatory effects have been described in the TRPV2^+/−^ rat model. In this model, several inflammatory mediators were upregulated, including IL-6, IL-13, IL-18, ICAM-1, and Ppbp, together with an increase in microglial cells. Interestingly, topical sitagliptin significantly reduced the expression of these inflammatory markers and decreased microglial density in the retina [18]. These findings indicate that sitagliptin is able to modulate different inflammatory pathways in distinct experimental conditions.

Therefore, topical sitagliptin shows consistent anti-inflammatory effects characterized by inhibition of the NF-κB pathway, reduction in pro-inflammatory cytokines, and downregulation of adhesion molecules. These actions, together with its previously described neuroprotective and antioxidant properties, support the potential of this therapeutic strategy in the early stages of retinal neurovascular impairment.

#### 4.4.4. Beneficial Effects of Sitagliptin Eyedrops on Glial Cells

Topical administration of sitagliptin has also been shown to exert relevant effects on retinal glial activation, which represents a key event in early neurovascular impairment [13,15,18]. Reactive gliosis is characterized by upregulation and redistribution of glial fibrillary acidic protein (GFAP), particularly in Müller cells, in which GFAP extends from the inner retinal layers throughout the entire Müller cell axis toward the outer retina. This abnormal pattern reflects glial stress and contributes to neuronal dysfunction [151,152].

Treatment with sitagliptin eyedrops was associated with attenuation of Müller cell gliosis in the db/db mouse. A reduction in GFAP expression was observed together with a more restricted distribution pattern, mainly confined to astrocytes in the nerve fiber layer, resembling the physiological condition [13,14,15]. The decreased extension of GFAP along Müller cell processes suggests partial restoration of glial homeostasis. Since Müller cells are central regulators of retinal metabolism, glutamate clearance, and inflammatory signaling, the reduction in their reactive state may have important functional implications. In the TRPV2^+/−^ model, sitagliptin also exerted multiple macroglial-modulatory effects. The retinal dysfunction of this model was associated with increased GFAP expression, together with a significant downregulation of Kir4.1 (*Kcnj10*), the principal inwardly rectifying potassium channel responsible for spatial K^+^ buffering and maintenance of glial ion homeostasis. Kir4.1 loss is a hallmark of impaired Müller cell function and contributes to altered extracellular potassium regulation, increased neuronal excitability, and progression of retinal stress [153]. Topical sitagliptin prevented both GFAP upregulation and Kir4.1 downregulation, suggesting preservation of Müller cell homeostatic properties and attenuation of reactive gliosis.

Beyond macroglial effects, sitagliptin also significantly influenced microglial activation in the same model. The retinas of the TRPV2^+/−^ rats showed increased microglial density across retinal layers, accompanied by an upregulation of inflammatory mediators. Treatment with sitagliptin reduced the number of Iba-1-positive microglial cells and promoted a morphology more consistent with a resting phenotype, characterized by smaller somata and more ramified processes [154]. Although not all cytokines were uniformly affected, the overall inflammatory profile was attenuated. These findings indicate that sitagliptin modulates both macroglial and microglial responses within a single neurovascular impairment model, limiting glia-driven inflammatory amplification and preserving structural and functional retinal homeostasis [18].

It is well established that activated Müller cells and microglia interact closely within the neurovascular unit. Müller cell gliosis promotes cytokine release and facilitates microglial activation, while activated microglia further stimulate macroglial reactivity, creating a self-perpetuating inflammatory cycle [155]. Therefore, the simultaneous reduction in GFAP expression and Iba-1-positive cells suggests that sitagliptin may interrupt this pathogenic loop. This effect could be partially explained by inhibition of the NF-κB pathway, which plays a central role in glial inflammatory signaling, and may also involve GLP-1-dependent mechanisms, since GLP-1 receptor activation has been associated with modulation of glial responses.

Importantly, attenuation of glial activation has been associated with preservation of retinal function [156]. The reduction in Müller cell gliosis and microglial activation correlates with improved electroretinographic responses and maintenance of retinal structural integrity (a topic discussed in more detail in one of the following chapters). In this context, the capacity of topical sitagliptin to limit both macroglial and microglial reactivity further supports its neuroprotective profile and its potential role in stabilizing the retinal neurovascular unit during early stages of retinal impairment.

#### 4.4.5. Vascular Improvement of Sitagliptin Eyedrops

Topical administration of sitagliptin has also been related to beneficial effects on retinal vascular integrity. Early vascular alterations are characterized by an increased vascular permeability, BRB breakdown, and a progressive microvascular degeneration, which precede the appearance of evident vascular lesions [44].

Treatment with sitagliptin eyedrops was associated with a significant reduction in vascular leakage [13,15]. This effect indicates preservation of the inner BRB and suggests improved endothelial function. Since vascular permeability has been intimately associated with inflammatory signaling and oxidative stress, the reduction in vascular leakage is consistent with the anti-inflammatory and antioxidant properties previously described for sitagliptin. Moreover, stabilization of the barrier may contribute to reducing secondary neuronal and glial damage. In the same line, in vitro experiments in both RPE and endothelial cells, which correspond to inner and outer BRB, revealed that sitagliptin was able to prevent hyperpermeability and BRB impairment [112]. However, Jäckle et al. evidenced in another in vitro study that sitagliptin was not able to prevent the destabilization of the BRB when endothelial cells were exposed to high VEGF levels [157].

Regarding the retinal microvascular structure, sitagliptin treatment reduced the formation of acellular capillaries in the TRPV2^+/−^ rat model. Acellular capillaries are the result of endothelial cell loss and impaired vascular maintenance; their appearance is a hallmark of early capillary degeneration, and it is considered a key indicator of irreversible microvascular damage [18,158]. The decreased number of acellular capillaries observed after sitagliptin administration in the TRPV2^+/−^ model suggests preservation of endothelial cell viability and improved vascular stability.

These vascular protective effects may be mediated by several mechanisms. The inhibition of inflammatory pathways, particularly the NF-κB signaling cascade, is known to reduce endothelial activation and leukocyte adhesion [34]. In this regard, the downregulation of adhesion molecules such as VCAM-1 after sitagliptin treatment may limit leukostasis and subsequent endothelial injury. Additionally, the antioxidant properties of sitagliptin may further protect endothelial cells from dysfunction and apoptosis [112].

As has already been mentioned, endothelial cells, glial cells, and neurons interact closely within the retinal neurovascular unit. Vascular leakage promotes glial activation and neuronal dysfunction, while reactive glia and activated microglia further exacerbate vascular damage through cytokine release [6,7,159]. Therefore, the vascular protective effects of sitagliptin are likely reinforced by its capacity to modulate inflammation and glial reactivity. This integrated action may contribute to breaking the vicious cycle linking vascular dysfunction and neuroinflammation. However, the strong interplay between these functional connections makes it difficult to determine whether the vascular protective effects are fully, partly, or not at all due to a direct action of sitagliptin on the blood vessels, or if they result indirectly from improvements in neuronal and/or glial function.

In this context, the vascular effects of topical sitagliptin complement its neuroprotective and anti-inflammatory actions and further support its potential to stabilize the retinal neurovascular unit during early stages of DRD.

#### 4.4.6. Improvement of Global Ocular Functional and Structural Outcomes

Topical sitagliptin has demonstrated its capacity to preserve retinal function and structure in early DRD in different animal models [13,16,18]. Electrophysiological assessment by full-field ERG showed that diabetes-induced functional impairment is attenuated by the treatment, particularly through the preservation of the a-wave amplitudes, which indicates maintenance of photoreceptor functionality. Notably, these improvements occur independently of any blood glycemic improvement, thus supporting a direct ocular effect of the topical formulation [13,16].

On the other hand, optical coherence tomography (OCT) revealed a significant prevention of retinal thinning, a hallmark of early retinal disease. Eyes treated with sitagliptin eyedrops present retinal thickness values closer to non-diabetic controls, indicating preservation of overall retinal architecture [18]. In parallel, fundoscopic evaluation showed attenuation of abnormal retinal vessel dilation and a reduction in early microvascular degenerative features (acellular capillaries) [18]. Taken together, the available evidence indicates that topical sitagliptin stabilizes the global ocular status in early DRD, with consistent functional and structural benefits detectable by clinically translatable techniques, such as ERG and OCT.

## 5. Ocular and Plasma Pharmacokinetics of Sitagliptin Eyedrops

Topical sitagliptin has been pharmacokinetically evaluated to determine whether therapeutically relevant concentrations can be achieved in the posterior segment after ocular administration. In rabbits, a single-dose instillation of sitagliptin eyedrops (concentrations of 5 mg/mL and 10 mg/mL) was followed by a quantitative analysis of plasma and ocular tissues, including iris/ciliary body, aqueous humor, vitreous humor, and retina/choroid, using a validated LC–MS/MS method. The measurements demonstrated rapid drug exposure in the retina/choroid tissue shortly after administration. Retinal concentrations increased in a dose-dependent manner, with the 10 mg/mL formulation achieving proportionally higher tissue levels than the 5 mg/mL concentration [105].

Importantly, systemic exposure remained minimal and negligible [105]. Plasma concentrations were below the range associated with systemic DPP-4 inhibition after oral administration, and they declined rapidly over time [105,160,161]. The results indicate local distribution of the sitagliptin and support the safety profile of the therapeutic approach. In parallel, sitagliptin levels detected in the aqueous and vitreous humors were comparatively low and did not parallel the early retinal detection. Retinal exposure was observed at time points when vitreous concentrations were still minimal, suggesting that diffusion through the anterior chamber and subsequent transvitreal transport does not represent the primary route of posterior segment delivery [105].

## 6. Limitations and Translational Challenges

Despite the promising results obtained in experimental models at the preclinical level, several limitations should be acknowledged. First, the current available evidence is limited, and most of the studies evaluating topical administration of sitagliptin come from a small number of research groups, highlighting the need for independent replication. In addition, this approach has been evaluated in a small number of experimental models, with most of its efficacy data derived from the db/db mouse model. Differences in outcome measures also complicate direct comparisons across studies. Furthermore, the exact cellular mechanisms underlying the beneficial effects of topical DPP-4 inhibition, including the differentiation between dependent and independent GLP-1 receptor pathways, require further investigation. Studies aimed at evaluating long-term ocular safety are also required before phase II clinical trials can be considered.

## 7. Conclusions

Due to the important role of neurodegeneration during the earliest stages of DRD, neuroprotective strategies have emerged as promising approaches. Among them, the inhibition of the enzyme DPP-4 has provided consistent and robust results in different experimental models.

**Figure 1:**
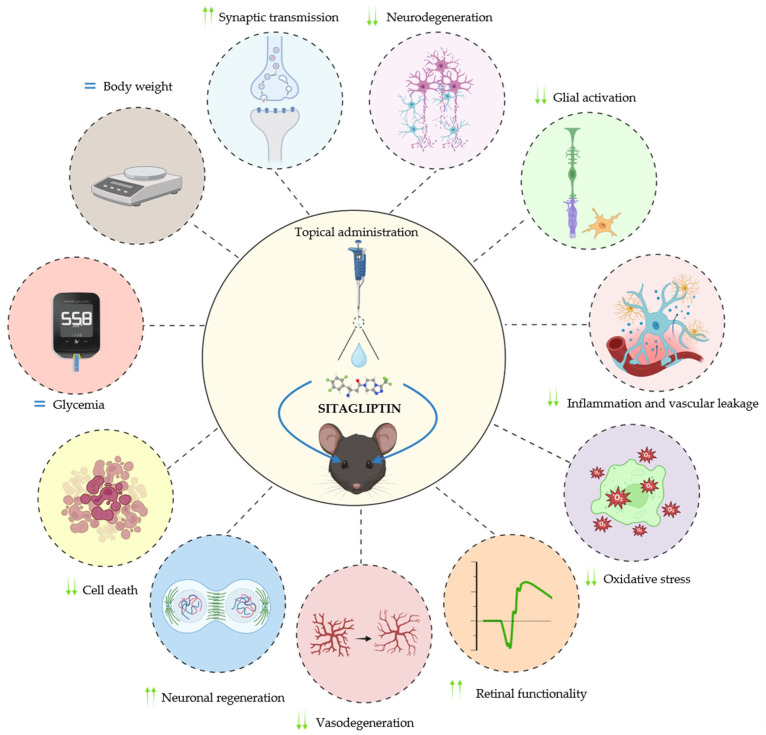
Summary of the main neuroprotective effects of sitagliptin eyedrops in two different animal models of early diabetic retinal disease (DRD). DPP-4 inhibition reduces oxidative stress, inflammation, and glial activation while preserving synaptic machinery and the integrity of the blood–retinal barrier (BRB), resulting in an attenuation of cell death. These effects occur without altering body weight or glycemia. Overall, they contribute to the stabilization of the retinal neurovascular unit (NVU) and subsequent functional and structural improvements. Attenuation of acellular capillary formation was only observed in the TRPV2KO rat model.

Topical administration of DPP-4i, more specifically, administration of sitagliptin eyedrops, has shown neuroprotective, anti-inflammatory, antioxidant, and vasculoprotective effects at the preclinical level. These myriad of mechanisms of action results in the preservation of synaptic integrity, attenuation of the oxidative and inflammatory state, reduction in glial activation, improved BRB function with the consequent diminishment of vascular leakage, and decreased formation of acellular capillaries, contributing to stabilization of the retinal neurovascular unit and functional and structural improvements of DRD and other diseases in which neurodegeneration plays an essential role, such as glaucoma (Figure 1). Notably, this approach enables direct retinal action of the treatment while minimizing systemic exposure. Overall, sitagliptin eyedrops represent a promising non-invasive strategy for treating retinal neurodegeneration.

However, a limitation of this approach is the lack of extensive experimental validation beyond our own studies, as most of the available preclinical evidence has been generated by our research group. In any event, clinical studies are necessary to confirm their efficacy and their long-term ocular safety.

## 8. Patents

Two of the authors (Cristina Hernández and Rafael Simó) are inventors of the patent PCT/EP2017/060234, which is related to the use of dipeptidyl peptidase-4 inhibitors (sitagliptin) for topical eye treatment of retinal neurodegenerative diseases.

## Data Availability

No new data were created or analyzed in this study. Data sharing is not applicable to this article.

## Abbreviations

The following abbreviations are used in this manuscript:

AD	Alzheimer’s disease
AGE	Advanced glycation end-product
AMPA	α-amino-3-hydroxy-5-methyl-4-isoxazolepropionic acid
ARE	Antioxidant response element
ATP	Adenosine triphosphate
BBB	Blood–brain barrier
BDNF	Brain-derived neurotrophic factor
BRB	Blood–retinal barrier
CASK	Calcium-/calmodulin-dependent serine protein kinase
CAT	Catalase
cAMP	Cyclic adenosine monophosphate
CCL5	Chemokine (C-C motif) ligand 5
CNS	Central nervous system
CNTF	Ciliary neurotrophic factor
Dlg2	Discs large homolog 2
Dlg4	Discs large homolog 4 (PSD-95)
DPP-4	Dipeptidyl peptidase-4
DPP-4i	Dipeptidyl peptidase-4 inhibitors
DRD	Diabetic retinal disease
EMA	European Medicines Agency
EPO	Erythropoietin
ERG	Electroretinography
ERK	Extracellular signal-regulated kinase
FDA	Food and Drug Administration
GABA	Gamma-aminobutyric acid
GFAP	Glial fibrillary acidic protein
GIP	Gastric inhibitory polypeptide
GLP-1	Glucagon-like peptide-1
GLP-1R	Glucagon-like peptide-1 receptor
GPX	Glutathione peroxidase
GR	Glutathione reductase
GSEA	Gene set enrichment analysis
ICAM-1	Intercellular adhesion molecule 1
IL-1β	Interleukin-1 beta
IL-6	Interleukin-6
IL-13	Interleukin-13
IL-18	Interleukin-18
IRBP	Interstitial retinol-binding protein
JAK	Janus kinase
KBP	KIF1-binding protein
KIF1B	Kinesin family member 1B
NMDA	N-methyl-D-aspartate
NOX	NADPH oxidase
NOX2	NADPH oxidase 2
NADPH	Nicotinamide adenine dinucleotide phosphate
NF-κB	Nuclear factor kappa B
NGF	Nerve growth factor
NRF2	Nuclear factor erythroid 2-related factor 2
NVU	Neurovascular unit
OCT	Optical coherence tomography
PD	Parkinson’s disease
PEDF	Pigment epithelium-derived factor
PI3K	Phosphoinositide 3-kinase
PKC	Protein kinase C
PKC-β	Protein kinase C beta
PKC-δ	Protein kinase C delta
PPAR-α	Peroxisome proliferator-activated receptor alpha
Ppbp	Pro-platelet basic protein
PYY	Peptide YY
ROS	Reactive oxygen species
RPE	Retinal pigment epithelium
RT-qPCR	Reverse transcription quantitative polymerase chain reaction
SDF-1	Stromal cell-derived factor 1
SNAP-25	Synaptosomal-associated protein 25
SNARE	Soluble NSF attachment protein receptor
SOD	Superoxide dismutase
SOCS1	Suppressor of cytokine signaling 1
SST	Somatostatin
STAT	Signal transducer and activator of transcription
SV2B	Synaptic vesicle glycoprotein 2B
TNF-α	Tumor necrosis factor alpha
TRPV2	Transient receptor potential vanilloid 2
TXNIP	Thioredoxin-interacting protein
VCAM-1	Vascular cell adhesion molecule 1
VEGF	Vascular endothelial growth factor
VGLUT1	Vesicular glutamate transporter 1
